# Peroxisomes during postnatal development of mouse endocrine and exocrine pancreas display cell-type- and stage-specific protein composition

**DOI:** 10.1007/s00441-023-03766-6

**Published:** 2023-05-01

**Authors:** Claudia Colasante, Rocio Bonilla-Martinez, Timm Berg, Anita Windhorst, Eveline Baumgart-Vogt

**Affiliations:** 1 Institute for Anatomy and Cell Biology, Medical Cell Biology, Justus Liebig -University, Aulweg 123, 35392 Giessen, Germany; 2 Institute for Medical Informatic, Justus Liebig University, Rudolf-Buchheim-Str. 6, 35392 Gießen, Germany

**Keywords:** Peroxisomes, Exocrine pancreas, Endocrine pancreas, Postnatal development, Catalase

## Abstract

Peroxisomal dysfunction unhinges cellular metabolism by causing the accumulation of toxic metabolic intermediates (e.g. reactive oxygen species, very -chain fatty acids, phytanic acid or eicosanoids) and the depletion of important lipid products (e.g. plasmalogens, polyunsaturated fatty acids), leading to various proinflammatory and devastating pathophysiological conditions like metabolic syndrome and age-related diseases including diabetes. Because the peroxisomal antioxidative marker enzyme catalase is low abundant in Langerhans islet cells, peroxisomes were considered scarcely present in the endocrine pancreas. Recently, studies demonstrated that the peroxisomal metabolism is relevant for pancreatic cell functionality. During the postnatal period, significant changes occur in the cell structure and the metabolism to trigger the final maturation of the pancreas, including cell proliferation, regulation of energy metabolism, and activation of signalling pathways. Our aim in this study was to (i) morphometrically analyse the density of peroxisomes in mouse endocrine versus exocrine pancreas and (ii) investigate how the distribution and the abundance of peroxisomal proteins involved in biogenesis, antioxidative defence and fatty acid metabolism change during pancreatic maturation in the postnatal period. Our results prove that endocrine and exocrine pancreatic cells contain high amounts of peroxisomes with heterogeneous protein content indicating that distinct endocrine and exocrine cell types require a specific set of peroxisomal proteins depending on their individual physiological functions. We further show that significant postnatal changes occur in the peroxisomal compartment of different pancreatic cells that are most probably relevant for the metabolic maturation and differentiation of the pancreas during the development from birth to adulthood.

**Supplementary Information:**

The online version contains supplementary material available at 10.1007/s00441-023-03766-6.

## Introduction

The mammalian pancreas is a heterogeneous organ composed of exocrine and endocrine cells. Exocrine acini constitute around 95% of the pancreatic mass and produce digestive enzymes, which are excreted into the duodenum via the duct system. Endocrine cells are organised in well capillarised globular clusters known as islets of Langerhans, dispersed throughout the exocrine tissue especially towards the pancreas tail. The islets contain various cell types, each producing and secreting different peptide hormones: α-cells produce glucagon, β-cells insulin, δ-cells somatostatin and PP-cells pancreatic polypeptide. In mice, within the islets, β-cells constitute the majority (60–80%), forming a core around which the other cells are arranged (Murtaugh et al. 2003).

Pancreatic cells were long believed to contain only very few peroxisomes since the antioxidative enzyme catalase formerly used as primary marker for the identification of peroxisomes in biochemical assays or microscopic analysis is low abundant in this organ (Morikawa and Harada 1969; Hand 1974). Especially isolated pancreatic β-cells and RINm5F insulinoma cells were shown to contain less than 2% of catalase activity compared to liver tissue (Tiedge et al. 1997). Therefore, pancreatic β-cells were predicted to be more susceptible to oxidative stress (Grankvist et al. 1981; Tiedge et al. 1997; Robertson et al. 2003). Despite the low level of catalase, we previously demonstrated the presence of a considerable number of peroxisomes in pancreatic islets as well as in pancreatic exocrine tissue using antibodies against the peroxisomal biogenesis proteins PEX14 and PEX3 as well as against the lipid transporter ABCD3 (Grant et al. 2013; Colasante et al. 2017). Furthermore, a recent publication demonstrated that the β-cell-specific PEX5-knockout caused increased apoptosis and reduced the capability to secrete insulin, stressing the importance of the peroxisomal compartment for pancreatic β-cells (Baboota et al. 2019).

Generally, peroxisomes are intimately involved in lipid as well as ROS metabolism, whereby ROS degradation is not only accomplished through catalase, but also via multiple other enzymes including Cu/Zn-superoxide dismutase (SOD1) and peroxiredoxin 1 and 5 as well as a specific isoform of glutathione peroxidase (Wanders and Waterham 2006; Fransen et al. 2012; Islinger et al. 2018; Okumoto et al. 2020; Carmichael et al. 2022). Furthermore, they are highly dynamic organelles that rapidly adapt their metabolic activity in response to dietary changes, alterations in oxidative stress levels as well as treatment with hypolipidemic agents (Titorenko and Rachubinski 2001; Schrader and Fahimi 2006; Hettema and Motley 2009; Okumoto et al. 2020; Zalckvar and Schuldiner 2022). During late foetal and postnatal development the abundance and protein content of peroxisomes in mouse liver and brain as well as in differentiating human hepatoma cells are specifically regulated (Stier et al. 1998; Huyghe et al. 2001; Ahlemeyer et al. 2007).

As studied in several species including humans, rats, pigs, mice and ruminants, the post-natal development of the gastrointestinal tract and associated glands is triggered after birth by the switch to milk feeding and later by weaning (Pierzynowski et al. 1993; Abrahamse et al. 2012; Pluske 2013; Stolovich-Rain et al. 2015; Khan et al. 2016; Meale et al. 2017; Li et al. 2018). Being centrally involved in digestion and nutrient homeostasis, the pancreas is thoroughly affected by these dietary changes (Iovanna et al. 1990; Stolovich-Rain et al. 2015; Bonner-Weir et al. 2016). Many studies have contributed to our understanding of the adaptations that occur during the postnatal development of mammalian pancreas. These adaptations have been in part associated to adjustments of the enzymatic repertoire of glucose and mitochondrial energy metabolism and inner membrane metabolite transport (Farfari et al. 2000; MacDonald et al. 2005; Jitrapakdee et al. 2010; Jermendy et al. 2011). However, to date to our knowledge, nothing is known about the alterations of the peroxisomal compartment and its enzyme composition during the postnatal development of the pancreas. Therefore, in this study, we present a comprehensive investigation on the heterogeneity of the peroxisomal enzyme composition in distinct cell types of the exocrine and endocrine pancreas and its specific alterations during murine postnatal development.

## Materials and methods

### Isolation of protein from pancreatic and hepatic tissue

To isolate peroxisome-enriched fractions from the pancreas and liver, 3 wild-type C57BL/6J adult mice were killed through cervical dislocation after isoflurane (4.5%) anaesthesia and immediately perfused with 1 × PBS buffer through the left ventricle. Approximately 50 mg of pancreas and liver were removed and placed into a tube containing 1 ml cold lysis buffer (0.1% Triton X-100, 150 mM NaCl, 50 mM Tris pH 8.0) supplemented with 10% protease inhibitor mix (Serva). The tissue was then shredded using an ultra-turrax. The lysate was homogenised on ice with a Dounce homogeniser using 10 manual strokes. Nuclei were removed by centrifugation at 1,000 × *g* at 4 °C for 10 min followed by the removal of the mitochondrial enriched fraction at 5,000 × *g* at 4 °C for 10 min. The peroxisome-enriched fraction was collected at 30,000 × *g* and resuspended in 100 µl lysis buffer.

For whole pancreatic lysates proteins of wild type C57BL/6J mice aged P0.5, P15 and P84 (3 animals per age) were killed through cervical dislocation after isoflurane (4.5%) anaesthesia and immediately perfused with 1 × PBS buffer through the left ventricle. Approximately 50 mg of pancreas (P15 and P84) and liver (P84) were removed and placed into a tube containing 1 ml cold lysis buffer (0.1% Triton X-100, 150 mM NaCl, 50 mM Tris pH 8.0) supplemented with 10% protease inhibitor mix (Serva). For P0.5 pancreata, 30 mg of tissue and 500 µl of lysis buffer were used. The lysate was homogenised on ice with a Dounce homogeniser using 10 manual strokes. Cell rests were removed by centrifugation at 500 × *g* at 4 °C for 10 min.

The protein concentration was determined using the Bradford assay (Bio-Rad) according to manufacturer’s protocol. Samples were frozen at − 80 °C until western blot was performed.

### Western blot analysis

For western blotting, 10 µg enriched peroxisomal fraction or 40 µg tissue lysate were loaded on a 12% SDS-PAGE. Proteins were separated for 1 h at 200 V using 1 × gel running buffer (25 mM Tris, 192 mM glycine, 0.1% SDS). Tank blotting (Bio-Rad) was performed for 1 h at 100 V in cooled Towbin buffer (20% methanol, 192 mM glycine, 25 mM Tris pH 8.3) onto a polyvinylidene fluoride membrane (Immobilion^®^-P Transfer Membrane, Millipore).

Membranes were blocked using 7.5% (w/v) non-fat dry milk in TBS-Tween (150 mM NaCl, 50 mM Tris-HCl, 0.2% (v/v) Tween 20, pH 7.6) for 30 min at room temperature with gentle shaking followed by incubation for 1 h with 7.5% milk TBS-Tween containing appropriately diluted primary antibody (Supplementary Table 1). Membranes were then washed once for 15 min and twice for 5 min in TBS-Tween, followed by incubation for 45 min at room temperature with appropriately diluted secondary antibody (Supplementary Table 2). Finally, the membranes were extensively washed once for 15 min and four times for 5 min in TBS-Tween and processed depending on the secondary antibody either using the ECL (Clarity^®^ Western ECL Substrate, Bio-Rad) or the alkaline phosphatase (Immun-Star™ AP Chemiluminescence, Bio-Rad) detection kit according to the manufacturer’s protocol. The blot was exposed to a CL-X Posure^®^ film (Thermo Scientific) and developed with Readymatic Developer and Fixer (Carestream). Coomassie Brilliant Blue (CBB) was used as loading control as the abundance of the metabolic enzyme GAPDH, frequently used as loading control, was subject to changes during mouse postnatal development (not shown). CBB staining of the membranes was performed using SimplyBlue SafeStain (Invitrogen) according to the manufacturer’s protocol.

### Indirect immunofluorescence staining of paraformaldehyde-fixed paraffin-embedded mouse pancreata

Wild-type C57BL/6J mice (3–4 months old) or P0.5, P15 and P84 old mice were used to investigate the abundance of peroxisomes in pancreatic tissue. The animals were killed through cervical dislocation after isoflurane (4.5%) anaesthesia and immediately perfusion-fixed with 4% paraformaldehyde (PFA) containing 2% saccharose in PBS (pH 7.4) injected through the left ventricle. After overnight fixation, the samples were paraffin-embedded using a Leica TP 1020 automated vacuum infiltration tissue processor. Using a Leica RM2135 rotation microtome, sections (2 μm and 5 μm) were cut and stored until needed. An indirect immunofluorescence protocol was established for the immunolabeling of mouse pancreatic paraffin sections. A first deparaffinization step was carried out in an oven at 50 °C overnight. After a second deparaffinization procedure by immersing the slides in Xylene 3 times for 10 min, the sections were rehydrated in ethanol at different dilutions (2 times 99%, 96%, 80%, 70%, 50%) 3 min each and finally rinsed in dH_2_O for 2 min. For antigen retrieval, the samples were treated first with trypsin (0.01%) in 1 × PBS for 8 min at 37 °C followed by microwave irradiation. Before microwaving, the samples were washed 3 times in 1 × PBS (5 min each) and once with dH_2_O. Thereafter, microwaving was carried out for 3 times 5 min in 10 mM sodium citrate buffer at pH 6 to allow the accessibility of epitopes. Subsequently, the samples were allowed to cool and treated with a series of washing steps (once in dH_2_O and 3 times 5 min in PBS). Non-specific binding sites were blocked with 4% BSA in 1 × PBS for 2 h at RT in a moist chamber to prevent drying. The sections were then washed in PBS and finally incubated with primary antibody (Supplementary Table 1) at the optimised dilution in 1% BSA in 1 × PBS containing 0.05% Tween 20 overnight in a humid box. The next day the specimen were rinsed 3 times in 1 × PBS for 5 min each and then incubated with the appropriate fluorochrome-conjugated secondary antibody (Supplementary Table 2) in dilution buffer for 2 h in the dark to prevent fluorochrome fading. Thereafter the slides were rinsed 3 times in 1 × PBS for 5 min each and then incubated for 10 min with DAPI diluted in 1 × PBS to visualise nuclei. Finally, coverslips were mounted with Mowiol 4–88 (Polysciences) and n-propyl gallate (Sigma-Aldrich) as anti-fading agent at a 3:1 dilution overnight at room temperature protected from light.

### Morphometry

Following the immunofluorescence analysis of PEX14 and catalase, images were taken using a Leica TCS SP2 confocal laser scanning microscopy with a 63 × oil objective and 12 × sampling. Images were quantified using the program Image-Pro Plus 4.5. Peroxisome abundance was determined using two different parameters: (i) peroxisome area in relation to the analysed area (region of interest: ROI) (Fig. 8a) and (ii) peroxisome number in relation to the number of nuclei (Fig. 8b). After selecting and sizing a region of interest, the number of and the area occupied by peroxisomes and the number of nuclei within the ROI were automatically determined by the Image-Pro Plus 4.5 programme.

### Statistical analysis

Statistical analyses were conducted using R version 3.4.1 (https://www.R-project.org/, R Core Team (2017)). Obtained numbers were first transformed using the logarithm to the base of ten to obtain normal distributed values. Initially, the number of peroxisomes in the exocrine and endocrine pancreas was compared between the different ages of the animals using a Kruskal-Wallis-Test. Then, the number of peroxisomes was analysed using a multifactorial analysis of variance (ANOVA) testing effects of age of the animals providing the cells, endocrine or exocrine origin of the cells and the interaction between those two factors. These effects were corrected for eventual differing layer thickness of the histological slices fitting a linear mixed-effects model with maximum likelihood estimator using the R-package *nlme* (version 3.1-131) (Pinheiro et al. 2006). Afterwards, effects between endocrine and exocrine at different ages and between the different ages in the endocrine and exocrine pancreas were compared in simultaneous tests for the general linear hypotheses. *P* values were adjusted for multiple testing using the false discovery rate (FDR). For the multiple comparison of effects, the R-packages *multcomp* (version 1.4-7) (Hothorn et al. 2008) and *lsmeans* (version 2.27-2) (Lenth 2016) were used.

## Results

### The abundance of peroxisomal proteins was considerably lower in pancreas compared to liver

**Fig. 1:**
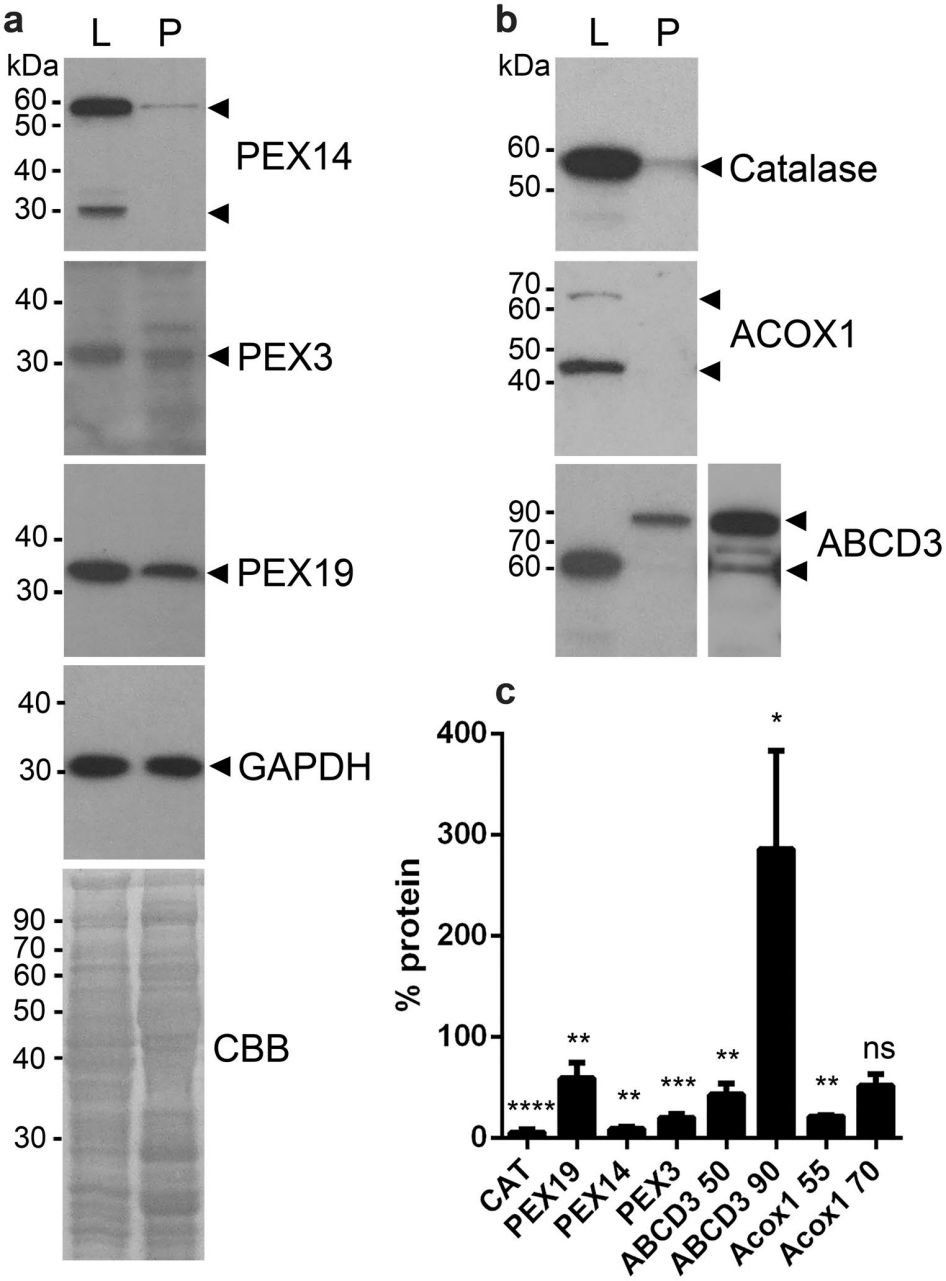
Western blot analysis of peroxisome-enriched fractions isolated from pancreas and liver. In each lane, 10 μg enriched peroxisomal fraction from liver (L) and pancreas (P) were loaded. Western blots were probed with antibodies against PEX14, PEX3, PEX19, ACOX1, ABCD3 and catalase (**a**, **b**). For the western blots shown in (**a**) and (**b**) GAPDH and the CBB-staining were used as loading control (**a**). Arrowheads are pointing to the specific protein bands corresponding to the used antibody (**a**, **b**). Protein band densities for the pancreas were calculated using image J and were plotted as percentage of the protein band densities detected in the liver (**c**)

In hepatocytes, peroxisomes and peroxisomal proteins are particularly high abundant, and therefore the liver was often used as reference and model tissue for peroxisomal studies. We compared the abundance of PEX14, PEX3, PEX19, ACOX1, ABCD3 and catalase in enriched peroxisomal fractions from the pancreas and liver derived from adult mice using western blot analysis. GAPDH and the CBB staining were used as loading control. All analysed proteins were less abundant in pancreas compared to the liver (especially catalase, ACOX1 and PEX14) (Fig. 1). Noteworthy is that ABCD3 in the pancreas presents different molecular weights than in the liver (Fig. 1). In the liver, ACOX1 displays two protein bands on the western blot analysis (Fig. 1). For ACOX1, the band of approximately 70 kDa corresponds to the full-length protein (A-band), and the one of 50 kDa is the B-band of the processed protein (Fig. 1) (Baumgart et al. 1996). In the pancreas, on the western blot analysis, ACOX1 was below detection limits (when loading 10 μg protein). The predicted molecular weight of PEX14 is 36 kDa, but in most analysed tissues and cell lines, it displays a size of 60 kDa on western blot analysis (Colasante et al. 2017). So far, no explanation exists in the literature for the observed size shift. The typical 60 kDa band can be detected in both the liver and the pancreas (Fig. 1). Additionally, in the liver, a protein band of approximately 30 kDa can be seen (Fig. 1).

### An optimised immunofluorescence protocol for pancreatic tissue allows the simultaneous detection of pancreatic hormones and peroxisomal membrane and matrix proteins

Our group has already described the best conditions for the immunofluorescence-based detection of peroxisomal antigens in many tissues (Grabenbauer et al. 2001; Grant et al. 2013; Colasante et al. 2017), but their localisation in murine pancreas required the adaptation of the existing protocol to allow equal detection efficiency for all tested antigens. For this reason, we have (i) tested different citrate buffer microwave boiling times (0, 5, 10 and 15 min) to improve antigen retrieval (Supplementary Fig. 1a–d), (ii) tried different FFPE tissue slice thicknesses (2 and 5 μm) (not shown) and (iii) optimised the antibody dilutions (not shown). For optimisation of the microwave boiling time for double immunofluorescence analysis, we have used the combination of antibodies against PEX14 and glucagon and tissue sections of 2 μm. Incubating the pancreatic tissue in citrate buffer at RT resulted in an excellent detection of glucagon but little signal for the peroxin PEX14. We found that increasing the boiling time improved the PEX14 signal intensity but decreased the glucagon staining. Very good staining for both PEX14 and glucagon was achieved by incubating the pancreatic tissue in boiling citrate buffer for 15 min (3 × 5 min microwaving) (Supplementary Fig. 1d).

### Peroxisomal enzymes, membrane transporters and peroxins are heterogeneously distributed in different cell types of the mouse pancreas

**Fig. 2:**
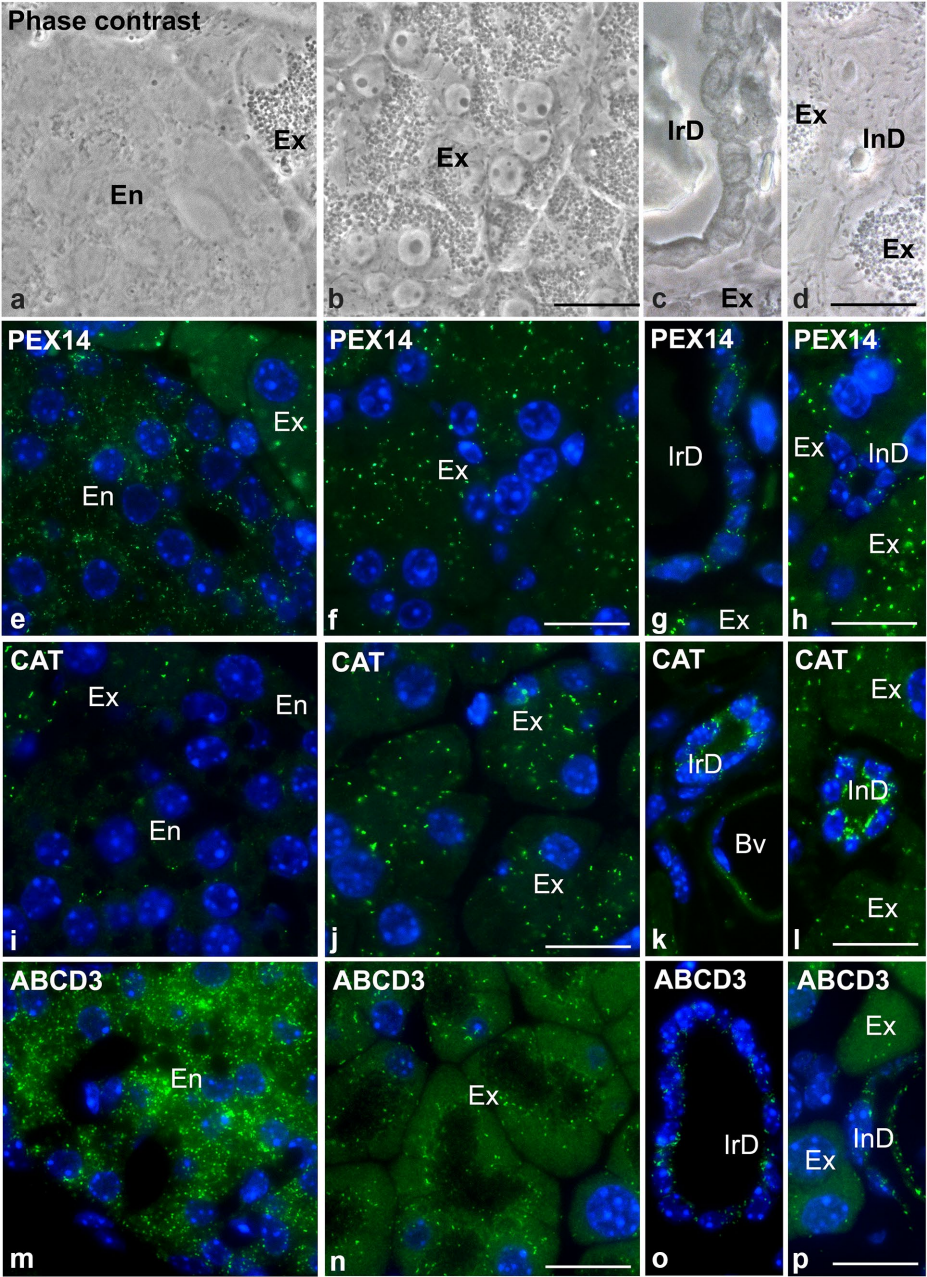
Abundance of PEX14, CAT, and ABCD3 in different regions of the pancreatic tissue. Phase contrast images (**a**–**d**) of tissue areas analysed in the PEX14-staining in (**e**)–(**h**). Immunofluorescence analysis of mouse pancreas using antibodies directed against PEX14 (**e**–**h**) catalase (**i**–**l**) and ABCD3 (**m**–**p**). Nuclei were counterstained with DAPI. **a**, **e**, **i, m** Representative picture of mainly endocrine cells of Langerhans islets; **b**, **f**, **j, n** exocrine acini clusters; **c**, **g**, **k**, **o** interlobular duct; **d**, **h**, **l**, **p** intralobular ducts. En, endocrine pancreas; Ex, exocrine pancreas; IrD, interlobular duct; InD, intralobular duct. Scale bar = 20 µm

We next investigated the distribution of several peroxisomal proteins in murine pancreatic tissue using immunofluorescence analysis. The peroxin PEX14 is part of the docking complex for the import of matrix proteins. Because of its homogeneous and ubiquitous distribution, it is an optimal marker protein for the detection of peroxisomes in the immunofluorescence analysis of tissue (Grant et al. 2013; Colasante et al. 2017). Figure 2a–d show phase contrast images of the analysed areas, namely endocrine (Fig. 2a) and exocrine (Fig. 2b) pancreatic cells and the interlobular (Fig. 2c) and intralobular ducts (Fig. 2d). In the endocrine cells, PEX14 is found in peroxisomes that are located around the nucleus (Fig. 2e). In the exocrine acini and secretory ducts, PEX14-labelled peroxisomes are mainly found in the apical part of the cells above the nucleus but underneath the zymogen granules facing the lumen (Fig. 2f–h). The apical localization of the peroxisomes is well demonstrated in the ABCD3 staining of a longitudinal section of an acinus shown in Supplementary Fig. 2. The immunofluorescence staining further revealed that PEX14 is less abundant in the epithelium of the interlobular and intralobular excretory ducts compared to the endocrine and exocrine cells (Fig. 2e, h). An overexposed image of the secretory ducts shown in Fig. 2e, h clearly confirms the presence of PEX14-stained peroxisomes in this part of the exocrine pancreas (Supplementary Fig. 3).

Immunofluorescence analysis showed that catalase staining is detectable in few individual cells of the endocrine pancreas (Fig. 2i). In contrast, catalase was abundant within peroxisomes in the epithelial cells of the exocrine acini (Fig. 2j). Catalase was particularly high abundant in the excretory ducts (Fig. 2k, l).

Unlike observed for most peroxisomal proteins, ABCD3, a peroxisomal transporter of branched- and long-chain fatty acids, was similarly abundant in individual peroxisomes of the exocrine and endocrine pancreas as well as in the interlobular ducts (Fig. 2m–p). The ABCD3-staining further suggests that peroxisome distribution per tissue area is less dense in the exocrine compared to the endocrine pancreas as confirmed by the morphometric experiments of the PEX14-staining shown in Fig. 8. The results of the qualitative quantification of the staining intensities of the peroxisomal proteins in endocrine and exocrine islets are summarized in Table 1.

**Table 1:** Qualitative assessment of the staining intensities in endocrine and exocrine islets

Pancreas	Endocrine	Exocrine	
Peroxisomal markers	All cell types	Acini	Ducts
PEX14	+++	+++	++
CAT	+	+++	+++
ABCD3	+++	++	+++

**Fig. 3:**
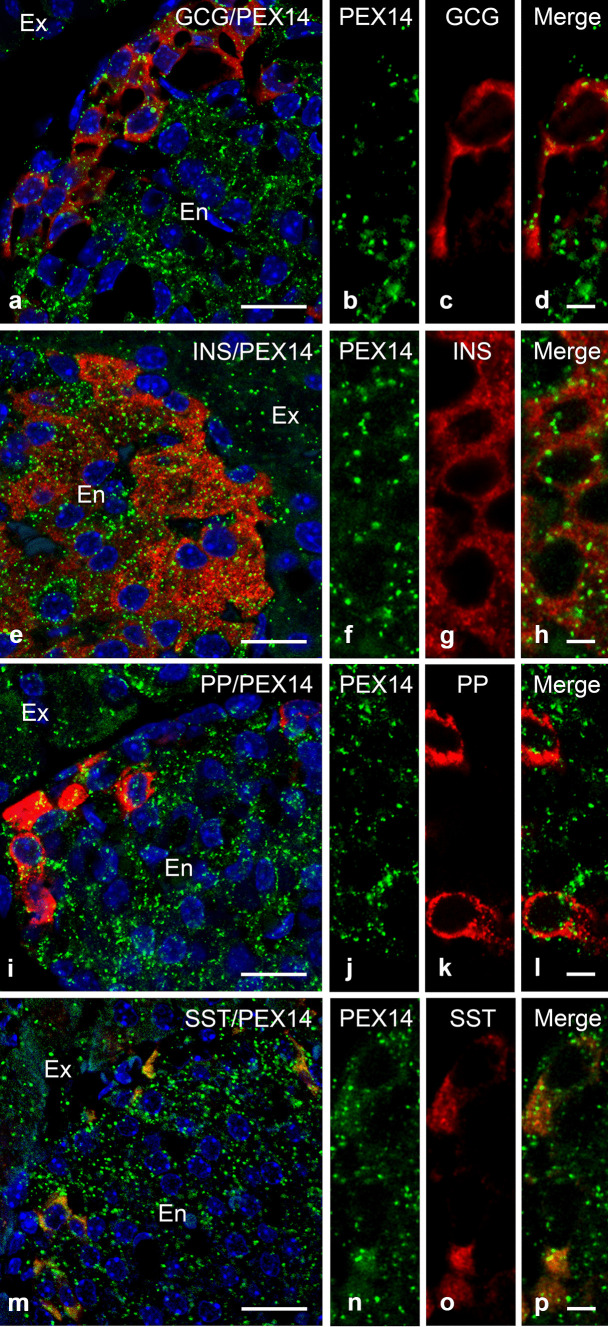
PEX14 positive peroxisomes show a heterogeneous distribution in α-, β-, PP- and ∂-cells. Double-immunofluorescence analysis of PEX14 and the peptide hormones glucagon (**a**–**d**), insulin (**e**–**h**), pancreatic polypeptide (**i**–**l**) and somatostatin (**m**–**p**) showing the abundance and localization of PEX14-labelled peroxisomes in different cell types of the islets of Langerhans. Higher magnifications of the images **a** (**b**–**d**), **e** (**f**–**h**), **i** (**j**–**l**) and **m** (**n**–**p**) respectively. Abbreviations: GCG, glucagon; INS, insulin; PP, pancreatic polypeptide; SST, somatostatin; PEX14, peroxisomal biogenesis protein 14; Ex, exocrine pancreas; En, endocrine pancreas. Scale bar in (**a**), (**e**), (**i**) and (**m**) = 25 μm. Scale bars in the correspondent higher magnifications images: 5 μm

In addition to the peroxisomal proteins, the peptide hormones glucagon, insulin, pancreatic polypeptide (PP) and somatostatin, which are produced in the islets of Langerhans, were analysed by immunofluorescence to visualise α-, β-, ∂- and PP-cells respectively (Fig. 3). The double labelling using antibodies directed against glucagon and PEX14 revealed visibly fewer PEX14-labelled peroxisomes in the α-cells, which are located at the outer rim of the islet, in comparison to the insulin-stained β-cells, which are located at the centre of the islets (Fig. 3a–h). Like for glucagon, the fluorescent signal for somatostatin (Fig. 3m) and pancreatic polypeptide (PP) (Fig. 3i) was detected in cells located in the outer rim of the islets. The overlay with PEX14 showed that peroxisomes were more abundant in ∂-cells (Fig. 3p) than in PP-cells (Fig. 3l). The staining intensity of PEX14 in PP-cells resembled the one observed in the α-cells. The cellular distribution of the peroxisomal proteins in the endocrine islets is summarized in Table 2.

**Table 2:** Qualitative summary of staining intensities in different endocrine islet cells assessed by immunofluorescence analysis in comparison to exocrine acini

	Exocrine	Endocrine			
	Acini	α-cells	β-cells	∂-cells	PP-cells
PEX14	+++	++	+++	+++	++

**Fig. 4:**
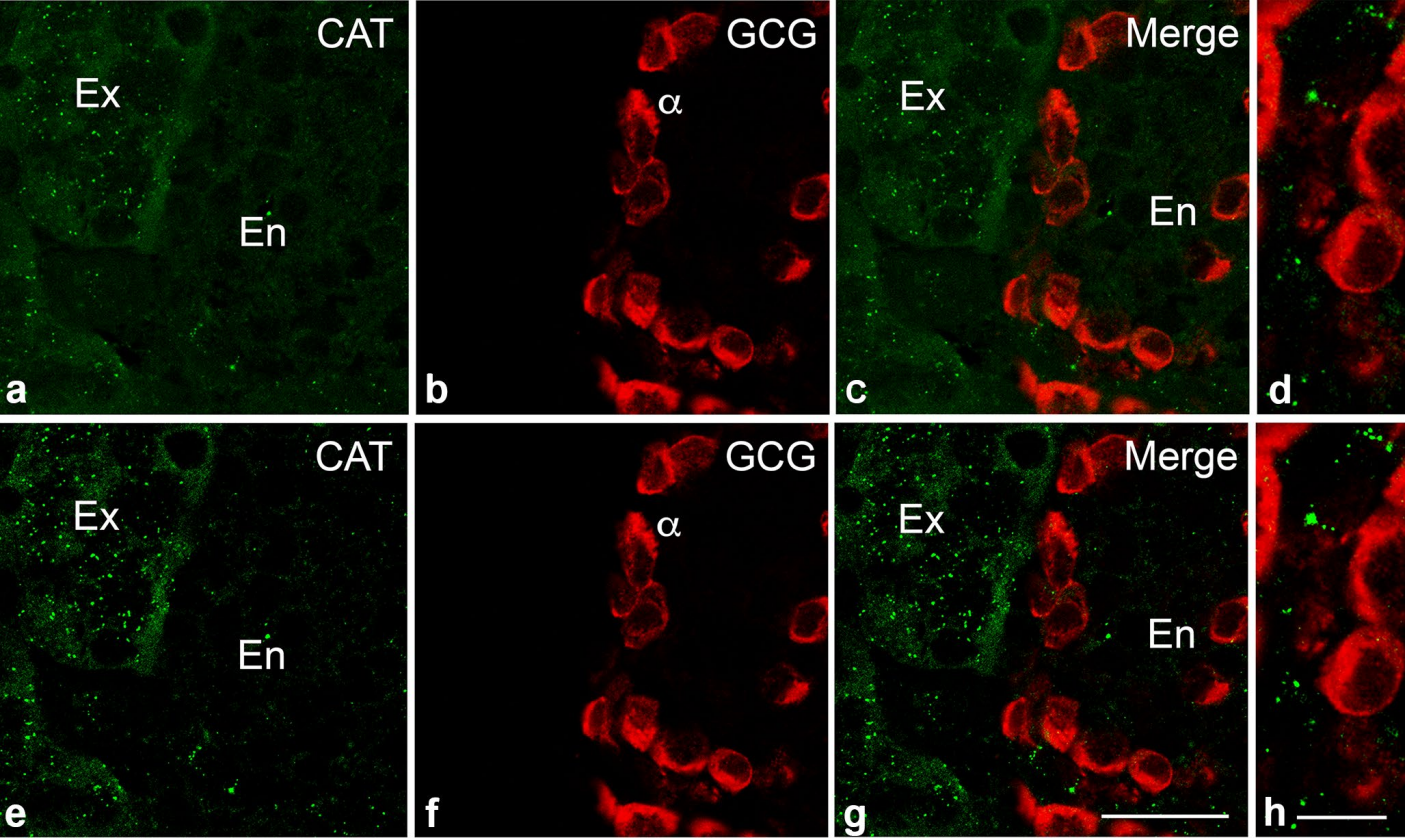
Immunofluorescence staining for glucagon and catalase in mouse pancreatic tissue. Analysis of the colocalization of GCG and CAT in α-cells (**a**–**d**). Higher magnifications depicting the distribution of CAT in peroxisomes of α-cells (**e**–**h**). Overexposure and magnification of image **c** (**d**) and **g** (**h**). For different antigen retrieval, see Supplementary Fig. 1. Abbreviations: GCG, glucagon; CAT, catalase; Ex, exocrine; En, endocrine. *Erythrocytes. Scale bar in (**a**–**c**) = 50 µm. Scale bar in the correspondent higher magnifications of the IF staining (**e**–**g**): 30 µm. Scale bar in (**d**) = 20 µm and (**h**) = 5 µm

A previous immunofluorescence analysis showed that α-cells contain very high amounts of cytosolic catalase compared to the other islet cells and exocrine acini (Bloch et al. 2006, 2007), but our results (Fig. 4) as well as previous publications (Grant et al. 2013) do not confirm this finding, suggesting non-specific labelling or cross-reactivity amongst the catalase and the glucagon antibodies used at that time.

### The amount of peroxisomal proteins in endocrine and exocrine pancreas varies during postnatal development

**Fig. 5:**
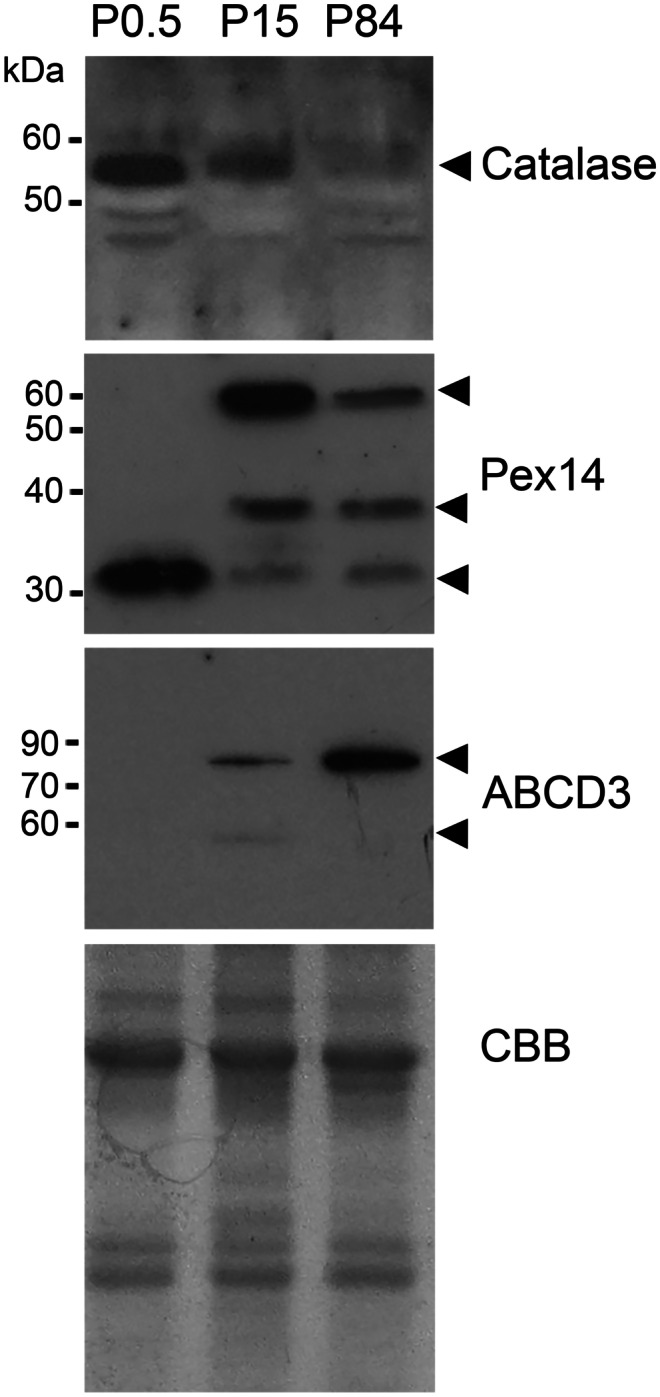
Abundance of catalase, PEX14 and ABCD3 in pancreas of mice during postnatal development. Pancreatic lysates (40 μg) from P0.5, P15 and P84 mice were used for western blot analysis and probed with antibodies against catalase, PEX14 and ABCD3. Arrowheads are pointing to the specific protein bands corresponding to the used antibody. As loading control, the membranes were stained with Brilliant Blue

**Fig. 6:**
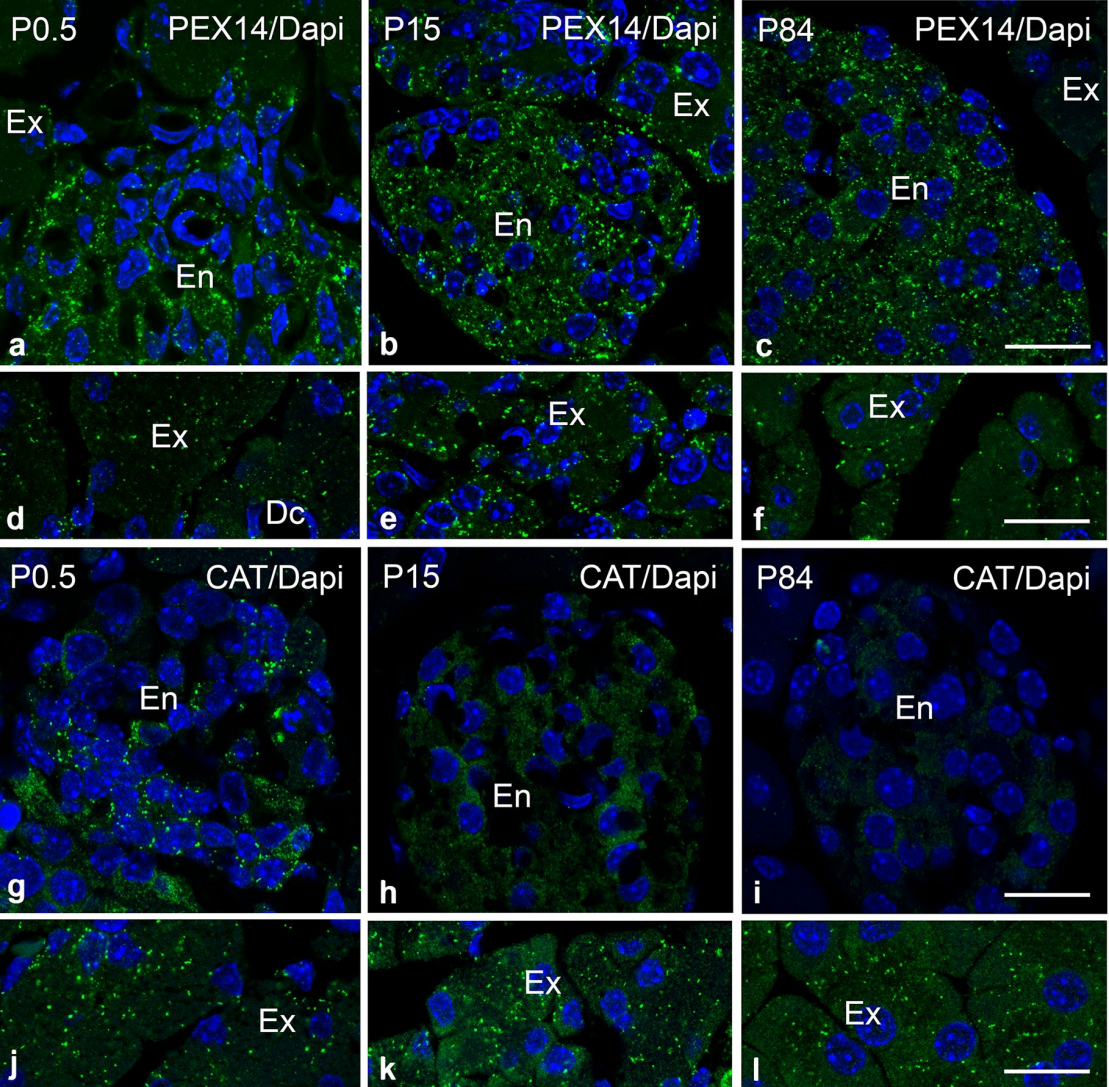
Distribution of PEX14 and catalase-positive peroxisomes in the exocrine and endocrine mouse pancreas during postnatal development. Distribution of PEX14-positive peroxisomes in endocrine pancreas (**a**–**c**) and in acinar cells of the exocrine pancreas (**d**–**f**) in P0.5, P15 and P84 mice. Catalase-stained peroxisomes in endocrine pancreatic tissue (**g**–**i**) and acinar cells (**j**–**l**) in P0.5, P15 and P84 mice. Nuclei were counterstained with DAPI. Abbreviations: Ex, exocrine; En, endocrine; Dc, duct. Scale bars = 30 μm

**Fig. 7:**
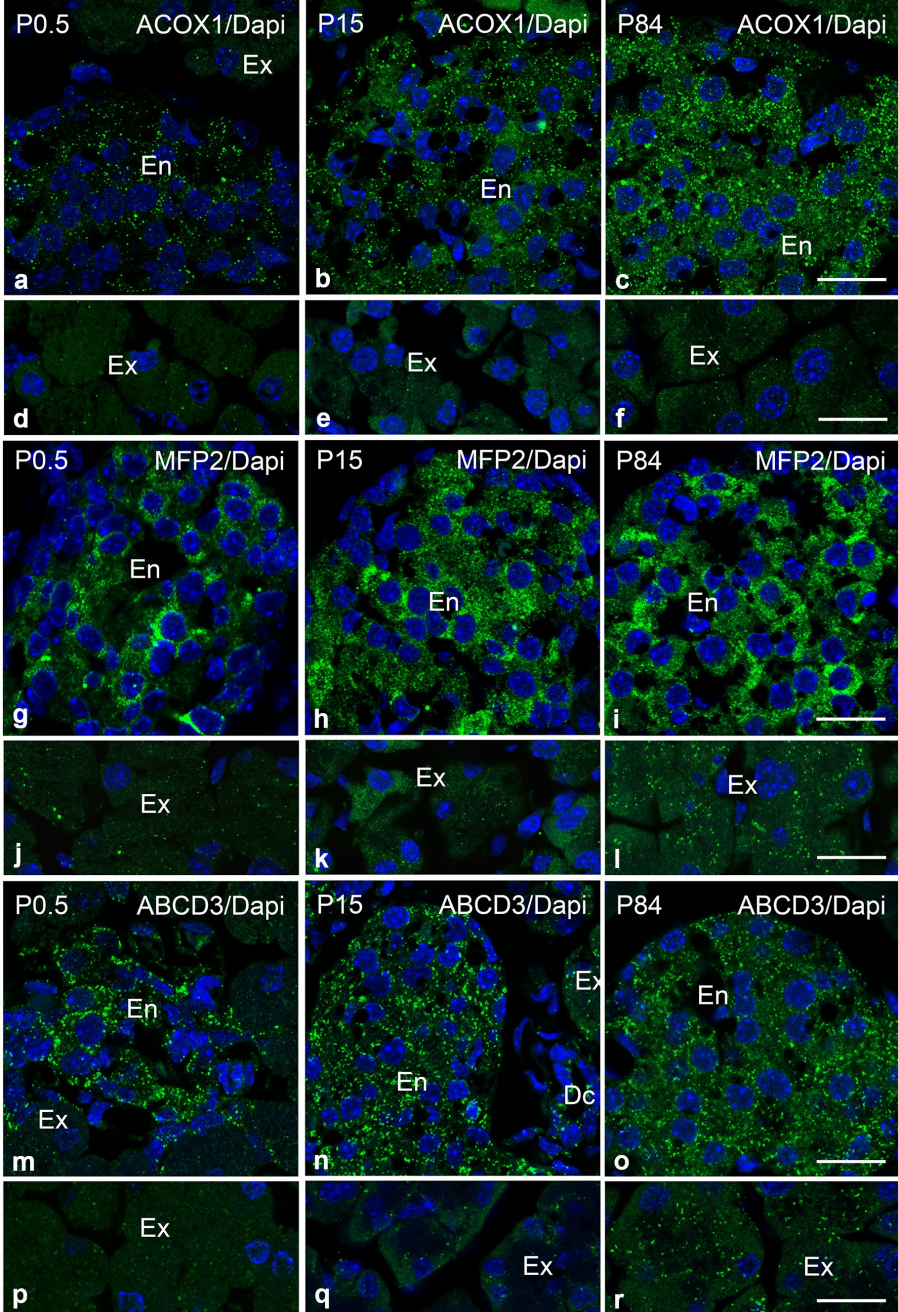
Distribution of ACOX1-, MFP2- and ABCD3-positive peroxisomes in the exocrine and endocrine mouse pancreas during postnatal development. **a–f** Immunofluorescence analysis of ACOX1 in the endocrine (**a**–**c**) and exocrine (**d**–**f**) pancreas of P0.5, P15 and P84 mice. **g**–**l** Immunofluorescence detection of MFP2 in endocrine (**g**–**i**) and exocrine (**j**–**l**) pancreas of P0.5, P15 and P84 mice. **m**–**r** Immunofluorescence detection of ABCD3 in endocrine (**m**–**o**) and exocrine (**p**–**r**) pancreas of P0.5, P15 and P84 mice. Nuclei were counterstained with DAPI. Abbreviations: Ex, exocrine; En, endocrine; Dc, duct. Scale bars = 30 μm

We next investigated the distribution, number and morphology of peroxisomes in endocrine and exocrine pancreas during murine postnatal development. To this purpose, we have performed western blot analyses (Fig. 5) and immunofluorescence staining (Figs. 6 and 7) using antibodies against several peroxisomal markers on pancreatic tissue derived from postnatal (P0.5), suckling (P15) and adult (P84) mice. Western blot analyses performed on whole pancreas lysates indicated age-related alterations in the protein abundance of catalase, PEX14 and ABCD3 (Fig. 5). Catalase, which was high-abundant shortly after birth (P0.5), was gradually reduced to very low levels until adulthood (P84) (Fig. 5). ABCD3, which was not detectable at P0.5, was most abundant at P84 (Fig. 5). Interestingly, the very weak band below 60 kDa observed at P15, and detectable in liver as well (Fig. 1), disappeared at P84, and only the prominent band between 70 and 90 kDa was visible (Fig. 5). Molecular weight shifts are also visible for PEX14, a typical observation in western blot analysis of this protein (Colasante et al. 2017). As previously mentioned, the commonly detected molecular size for PEX14 is approximately 60 kDa. This protein band was not present at P0.5, was strongly visible at P15 and became weaker at P84 (Fig. 5). The other bands detected at P0.5 and P15 were of approximately 32 kDa and 36 kDa, respectively. These 3 forms could possibly represent the monomer, a yet unknown modified monomeric form and a dimer of PEX14 (Fig. 5).

The immunofluorescence stainings with distinct peroxisomal antibodies further revealed differences in the age-related distribution of the investigated proteins in endocrine and exocrine pancreas (Figs. 6 and 7). Moreover, significant developmental alterations in acinar size and in islet organization were observed in P84 compared to P0.5 and P15 (Figs. 6 and 7). The results suggested that during postnatal development, the number of PEX14-stained peroxisomes was stable in the endocrine pancreas (Fig. 6a–c), while in the exocrine pancreas, the amount was lower at P0.5 and P84 compared to P15 (Fig. 6d–f). Similarly, we observed a peak in the catalase labelling intensity in exocrine pancreas at P15 (Fig. 6k). Catalase-containing peroxisomes in the islets of Langerhans could only be detected immediately after birth and at P15. However, catalase in peroxisomes became undetectable at P84 (Fig. 6g–i).

In contrast to catalase, the staining intensity for the β-oxidation enzymes ACOX1 (Fig. 7a–f) and MFP2 (Fig. 7g–l) increased with age in both endocrine an exocrine pancreas. The staining intensity for the lipid transporter ABCD3 visibly increased in the exocrine pancreas (Fig. 7p–r) when comparing P0.5 and P84 but displayed similar intensity in the endocrine pancreas throughout all analysed ages (Fig. 7m–o).

Inspection of the DAPI staining further indicated that exocrine pancreas at P15 contained a higher number of nuclei per ROI (Figs. 6e, k and 7e, k, q) compared to the other investigated ages and that at P84 the cytoplasm of the acinar cells was enlarged (Fig. 6f, l and 7f, l, r). These observations hint to cell proliferation and/or cell hypertrophy events occurring in the postnatal period as previously observed (Houbracken and Bouwens 2017).

### The observed differences in age-related abundance of PEX14 and catalase are statistically significant

To evaluate the robustness of the immunofluorescence analysis we have quantified the peroxisomal staining for PEX14 and catalase. PEX14 is the marker of choice for morphometrical analysis of peroxisomes as it is present in the docking complex for matrix protein import in every peroxisome and therefore allows the detection of differently sized peroxisomal subpopulations (Grant et al. 2013; Colasante et al. 2017). The morphometric results for PEX14 were compared to the morphometrical analysis of catalase-stained peroxisomes, which is the “historical” marker for peroxisomes (Fahimi 1969; Hirai 1969). During this analysis, the number of peroxisomes within a region of interest (ROI) and the number of peroxisomes compared to the number of nuclei per ROI were calculated. The latter value, e.g. peroxisome number per nucleus, is an indication for the number of peroxisomes per cell. This information is particularly important as mouse acinar cells of the pancreas are known to proliferate drastically in the first 4 weeks of life (Houbracken and Bouwens 2017).

In the following paragraphs, we first describe (a) the comparison of the *same* cell types (either endocrine or exocrine cells) at *different* time points during postnatal development and thereafter (b) the comparison of both cell types (endocrine vs. exocrine cells) at *identical* postnatal time points.

**Fig. 8:**
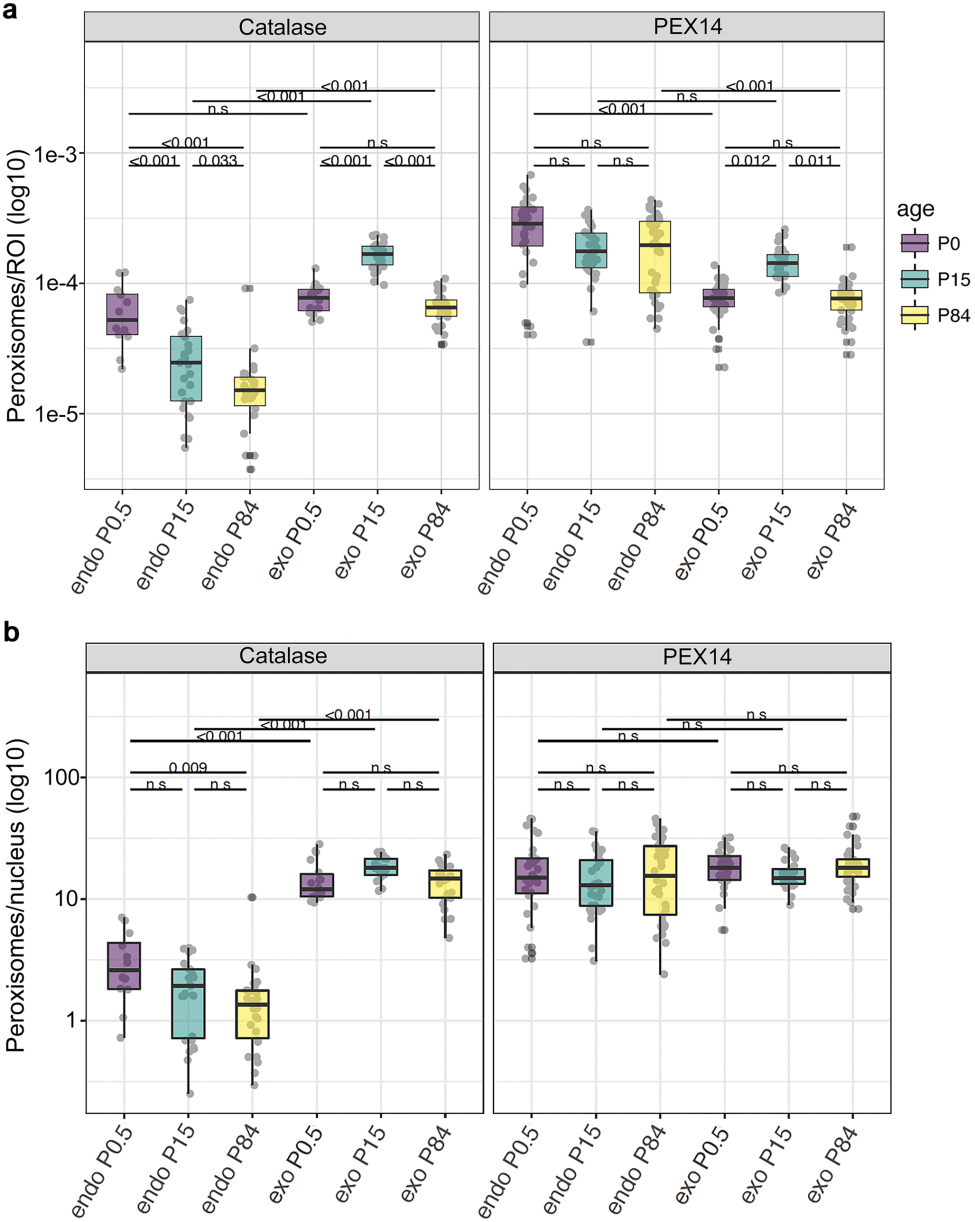
Morphometric analysis of catalase and PEX14-stained peroxisomes in exocrine and endocrine pancreas during postnatal development. Following the acquisition of fluorescence images, they were quantified using the program Image-J. **a** Peroxisome number in relation to the ROI. **b** Peroxisome number in relation to number of nuclei per ROI

To (a), in the *endocrine pancreas*, the number of catalase-stained peroxisomes per ROI as well as the number of catalase-stained peroxisomes per nucleus significantly decreased during postnatal development and was highest in new-born mice, suggesting that catalase abundance is downregulated during postnatal development in endocrine cells (Fig. 8a, b). In contrast, no changes in the number of PEX14-stained peroxisomes were observed (Fig. 8a, b).

In the *exocrine pancreas*, a significant increase in the number of catalase-containing peroxisomes per ROI was observed from P0.5 to P15, which dropped again thereafter (P84). In contrast, the number of catalase-labelled peroxisomes per nucleus did not vary during the entire time range. Similar morphometric changes were seen for the number of PEX14-stained peroxisomes in the exocrine pancreas (Fig. 8a, b). These results suggest that during postnatal development the number of peroxisomes per cell remains constant while the exocrine cells grow and are enlarged to their adult size.

To (b), the comparison of *endocrine vs. exocrine* cells, in newborn (P0.5) mice, the number of catalase-stained peroxisomes per ROI was not significantly different between endocrine and exocrine cells (Fig. 8a, b). However, significantly more catalase-positive peroxisomes per nucleus were counted in exocrine acinar cells in comparison to islet cells (Fig. 8a, b). At P15 and P84 the number of peroxisomes stained with catalase was always consistently higher in the exocrine acini than in islet cells (Fig. 8a, b). Moreover, a gradual decrease of catalase-positive peroxisomes was noted over time in the endocrine pancreas (Fig. 8a, b).

In contrast to catalase, the abundance of the PEX14-labelled peroxisomes per area was always higher in the cells of the islets of Langerhans regardless of the age of the animals. Furthermore, the number of peroxisomes per nucleus was consistently equal throughout all different time points in both exocrine and endocrine pancreas, suggesting the presence of more as well as smaller cells per ROI in the Langerhans islets in comparison to the larger exocrine acinar cells.

Thus, PEX14-stained peroxisomes are more abundant in the endocrine cells, while catalase-stained peroxisomes are more abundant in exocrine pancreas. Furthermore, an age-related decrease of the staining intensity of catalase exists in the endocrine pancreas.

## Discussion

In this paper, we demonstrated that endocrine and exocrine pancreatic tissues contain relatively high amounts of peroxisomes with variable protein content. We speculate that the different cell types found within the pancreas have specific requirements for peroxisomal metabolism and suggest that postnatal changes to the peroxisomal compartment are relevant for the maturation of pancreatic endocrine islets and exocrine acini.

### Heterogeneity of the peroxisomal population in the exocrine gland and the Langerhans islets as well as amongst their endocrine cell population in adult mice

One of the earliest and still commonly used cytochemical methods to identify peroxisomes in light and electron microscopy is the staining with 3,3′-diaminobenzidine (DAB) (Fahimi 1969; Hirai 1969; Novikoff and Goldfischer 1969) detecting the peroxidative activity of catalase (Fahimi 1969). Using this technique, Hruban et al. (1972) postulated the ubiquitous occurrence of peroxisomes in all vertebrate tissues. Due to its high protein abundance in peroxisomes of most tissues, catalase was therefore considered the marker of choice for detecting these organelles during morphological staining or western blot analyses of subcellular fractions (Grant et al. 2013; Colasante et al. 2017). In comparison to other tissues, e.g. liver, electron microscopy, immunohistochemistry, biochemical assays and northern blot analysis, it demonstrated that catalase activity and gene expression is relatively scarce in pancreatic endocrine and exocrine tissue, leading to the misconception that in this organ, peroxisomes are very low abundant (Morikawa and Harada 1969; Grankvist et al. 1981; Lenzen et al. 1996; Tiedge et al. 1997; Lei and Vatamaniuk 2011; Grant et al. 2013). In recent years, our laboratory established PEX14 as optimal marker for the immunohistochemical visualisation and the morphometric analysis of peroxisomes in mouse tissues and cell cultures (Grant et al. 2013; Colasante et al. 2017). This amply and ubiquitously expressed peroxisomal membrane protein is involved in matrix protein import and is readily accessible for antibodies due to its large cytoplasmic domain (Grant et al. 2013; Colasante et al. 2017). Using this marker and our optimized antigen retrieval conditions, PEX14-stained peroxisomes could readily and reproducibly be detected in both endocrine and exocrine adult pancreas confirming the presence of peroxisomes in all pancreatic cell types (Grant et al. 2013; Colasante et al. 2017).

In contrast to catalase, which is hardly present in cells of endocrine islets of adult animals, PEX14-labelled peroxisomes are highly abundant in the insulin producing β-cells and somatostatin-producing ∂-cells. This indicates that β- and ∂-cells possess an increased requirement for peroxisomes. Next to the specifically produced hormones, e.g. insulin, glucacon and somatostatin, differential expression of a variety of other genes was previously demonstrated during a transcriptome analysis using next generation Illumina based sequencing in β-, α- and ∂-cells (DiGruccio et al. 2016). When we screened the original data from this publication, we found that, despite their low expression, also some genes related to peroxisomal metabolism and biogenesis were differentially expressed in these islet cells. For example, the transcript counts for *Abcd3* were lower in ∂-cells compared to β- and α-cells. The counts for peroxins where generally equal in the three analysed cell types with exception of *Pex11*α and *Pex11*γ, and *Pex3*, which were lower in α-cells. Overall, α-cells appeared to contain slightly less counts for peroxisome-related genes especially catalase, which was also confirmed by our immunostainings in adult animals.

Our results on the abundance of peroxisomal proteins involved in lipid transport and β-oxidation are in complete agreement with the high transcript counts found for *Abcd3*, *Acox1*, and *Mfp2* in β-, α- and ∂-cells when compared to the ones of catalase (DiGruccio et al. 2016). Screening proteome data from isolated mouse islets (Petyuk et al. 2008) indeed revealed the presence of the peroxisomal lipid transporter ABCD3; the β-oxidation enzymes ACOX1, ACAA1A, ACAA1B, ACAA2, EHHADH and HSD17B4; the antioxidative enzyme PRDX5; and the peroxins PEX6, PEX14 and PEX19, in the pancreatic islets (Petyuk et al. 2008). Furthermore, a comparative proteome of single islets against whole pancreas revealed the elevated abundance of ABCD3 (4-fold), ACOX1 (4-fold) and MFP2 (2.8-fold) in the Langerhans islets (Waanders et al. 2009).

Heterogeneous distribution amongst pancreatic cell types was also observed for proteins involved in peroxisomal biogenesis: While PEX14-labelled peroxisomes were very high abundant in the endocrine islets (this study), we previously described that the peroxisomes labelled with PEX19 and PEX3 were more abundant in exocrine acini (Colasante et al. 2017). This differential distribution between endocrine and exocrine pancreatic cell types might be due to the specific functions of these proteins in distinct steps of peroxisomal biogenesis: (a) peroxisomal membrane biogenesis (PEX19 and PEX3) and (b) matrix protein import (PEX14). This difference is supported by a study from Waanders (Waanders et al. 2009) comparing the proteome of single islets to the one of complete pancreas in which PEX19 was 83-fold enriched in whole pancreas compared to islets (PEX3 was not classified).

In addition to the proteins that we analysed in this study, the proteomic analysis of Waanders et al. (2009) revealed that the peroxin PEX11β, the peroxisomal membrane transporter PMP34 and the acyl-CoA thioesterase 8 (ACOT8) were strongly enriched in islets. The peroxisomal proteins PEX1, sarcosine oxidase and peroxiredoxin 5 (PRDX5); the glycerolipid synthesising enzyme glycerophosphate acyltransferase (GNPAT); the α-oxidation enzyme phytanoyl-CoA dioxygenase (PHYH); and the β-oxidation enzymes trans-enoyl-CoA reductase (MFP1), trans-enoyl-CoA isomerase (PECI) and 3-ketoacyl-CoA thiolase (ACAA1a) were detectable at similar abundance within a 4-fold difference in both endocrine and whole pancreas (Waanders et al. 2009). Polyamine oxidase (PAOX) and PEX5 were moderately enriched in exocrine pancreas (Waanders et al. 2009).

In summary, pancreatic peroxisomes contain many enzymes involved in the metabolism of fatty acids and display exocrine/endocrine-specific composition. The high abundance of peroxisomal proteins involved in the metabolism of fatty acids is most likely because fatty acids play a crucial role in the regulation of the function of both pancreatic hormone and digestive enzyme production, storage and secretion (Bégin et al. 1990; Yaney and Corkey 2003; Danino et al. 2015; Acosta-Montaño and García-González 2018). Taken together, these results support the idea that the peroxisomal proteome and metabolism in highly differentiated cells are adapted to the individual cellular function.

### Heterogeneity of peroxisomes during postnatal development

The multiple organ defects and biochemical alterations seen in patients and corresponding knockout mouse models with generalized peroxisomal biogenesis disorders, such as cerebrohepatorenal syndrome (Zellweger syndrome), underscore that peroxisomes are essential for normal mammalian organ maturation (Li et al. 2002b; Baes and Van Veldhoven 2012; Braverman et al. 2016; Waterham et al. 2016; Wanders et al. 2018; Bose et al. 2022; Carmichael et al. 2022; Zalckvar and Schuldiner 2022).

Our results on the steady postnatal increment of proteins related to fatty acid metabolism indicate that during development, significant remodelling of the peroxisomal compartment occurs in the pancreas. A similar increase of activity and protein and transcript abundance of enzymes involved in fatty acid metabolism were previously observed in brain and liver (Lazo et al. 1991, Huyghe et al. 2001). In rat brain, the enzyme activity of ACOX1 was elevated at P16, decreased slightly after weaning and stabilized thereafter at a higher level than at P3 (Lazo et al. 1991). In mouse liver, transcription and protein abundance of α-oxidation and β-oxidation enzymes increased during the late foetal period and remained stable thereafter (Huyghe et al. 2001). For example, the protein abundance of ACOX1 and MFP2 visibly doubled at E18.5, suggesting that the increase of peroxisomal β-oxidation is coupled to early organ maturation (Huyghe et al. 2001). In an immunohistochemical study conducted in rats, the peroxisomal enzymes catalase, ACOX1 and thiolase were detectable from E11.5 in the liver and from E14.5 in the pancreas (Nardacci et al. 2004). Overall, the study investigated the daily abundance changes of catalase, ACOX1 and thiolase from E10.5 to E17.5 in 15 different rat organs and the CNS revealing that the increment of peroxisomal protein abundance during embryonal development is organ-, foetal stage- and protein-specific (Nardacci et al. 2004). This is not surprising as the differentiation of cells is accompanied by important changes to the peroxisomal compartment (Stier et al. 1998).

Postnatal organ development is influenced by nutritional changes, which necessitate rapid adaptation of energy and oxidative stress metabolism (Kaung 1994; Zabielski et al. 1998; Oštádalová and Babický 2012). These changes comprise the switch from carbohydrate (in utero) to high fat/low carbohydrate diet (milk) after birth (Henning 1981). The quick increase of the abundance of peroxisomal proteins involved in β-oxidation following birth can be explained by the necessity to cope with the increased amount of circulating fatty acids due to milk feeding. This suggests a central role of these peroxisomal pathways in cellular lipid homeostasis control. In the endocrine pancreas, the number of catalase positive peroxisomes decreased until P84, suggesting that decreasing the abundance of catalase in the endocrine pancreas in the postnatal period is relevant for the maturation of the hormone producing cells of the Langerhans islets. Immediately after birth, β-cells start proliferating (Scaglia et al. 1997) but do not yet display fully established glucose-sensitive insulin secretion and respond better to amino acids (Hellerström and Swenne 1991). Only the transition from high-fat milk to high-carbohydrate chow triggers a further maturation step that enhances glucose-stimulated insulin secretion and proliferation (Stolovich-Rain et al. 2015). Interestingly, experiments performed in embryonic rats revealed that hydrogen peroxide was important for the maturation of pancreatic β-cells and that the over-expression of catalase decreased the ability of β-cell to differentiate (Hoarau et al. 2014). Indeed, in β-cells catalase is considered by many authors a “disallowed” gene that interferes with their normal physiological function (Quintens et al. 2008; Pullen and Rutter 2013; Lemaire et al. 2016). The difference in catalase expression in exocrine and endocrine pancreas during the developmental period until adulthood is a further indication that, as already proposed in other publications (Yu et al. 1997; Huyghe et al. 2001; Herpin et al. 2003; Ahlemeyer et al. 2007; Colasante et al. 2015, 2017), peroxisomal maturation, differentiation and metabolism are strictly cell-type and tissue specific.

During postnatal development, the acinar population is first expanded through numerous mitotic divisions from preexisting acini during the suckling period (first and second week) (Houbracken and Bouwens 2017). Thereafter, the cells of the pancreatic acini grow through hypertrophy and display vastly enlarged cytoplasm at week 4. At this time point also the mitotic divisions are strongly reduced (Houbracken and Bouwens 2017). This is reflected by our observation that in the same exocrine pancreas area, more cells can be seen at P15 compared to P0 and P84, however, maintaining the same peroxisomal number. This indicates that in the mitotically dividing acinar cells of the P15 exocrine pancreas, (1) peroxisome number doubles before mitosis and individual organelles are then equally distributed to the daughter cells or (2) that the cells divide first and peroxisomes proliferated inside the “daughter” cells to reach the same numerical abundance as present in the “mother” cell. In yeast, peroxisomes divide before mitosis and are then divided evenly to the daughter cells (Hoepfner et al. 2001; Hettema and Motley 2009). In Hela and nH3T3 cells, peroxisomes are distributed evenly to the daughter cells (Kredel et al. 2009). How the different pancreatic cells maintain their various peroxisomal protein inventory and how this is regulated during the first weeks of postnatal development remain to be clarified in the future.

### Maintenance of cell-specific peroxisomal heterogeneity by PPARs

The metabolism and hormone secretion of endocrine pancreatic cells underlies complex and concerted regulation orchestrated by many factors: (i) nutritional state, (ii) nutritional composition, (iii) neurotransmitters, (iv) hormones and (v) paracrine secretion and signalling (Brunicardi et al. 1988; Hauge-Evans et al. 2009; Fu et al. 2013; Brereton et al. 2015; Briant et al. 2016). Peroxisomes are dynamic organelles that adapt fast to physiological changes such as oxidative stress as well as nutritional composition and fasting (Titorenko and Rachubinski 2001; Colasante et al. 2015, 2017; Kong et al. 2020). Important transcription factors of peroxisome-related genes are peroxisome proliferator-activated receptors (PPARα, -β and -γ). PPARs not only regulate peroxisome proliferation but exert a broad cellular response to control lipid and glucose homeostasis (Li et al. 2002a). In this respect, PPARs have also been reported to be important regulators of pancreatic cell homeostasis and influence the pathogenesis of both forms of diabetes (Charbonnel 2009; Huang et al. 2016; Holm et al. 2020; Frkic et al. 2021). PPARs display tissue specific distribution and in pancreas of adult rats; PPARβ mRNA was higher expressed than the one of PPARγ and PPARα (Braissant et al. 1996; Lemberger et al. 1996). Furthermore, the levels of the individual PPAR mRNAs were similar in the endocrine and exocrine pancreas (Braissant et al. 1996; Lemberger et al. 1996). In the pancreas, the activation of PPARγ has beneficial effects on mature β-cell function, inter alia through the activation of catalase in response to increased oxidative stress (Gupta et al. 2010, 2013; Kanda et al. 2010; Jetton et al. 2021). In the pancreas PPARα protects β-cells by activating peroxisomal β-oxidation in response to elevated fatty acids (Hellemans et al. 2007, 2009) or by promoting peroxisome proliferation and catalase activity in response to ROS (Yaribeygi et al. 2018). Depletion of PPARβ in mice increased pancreatic β-cell mass and improved insulin secretion causing hyperinsulinemia (Iglesias et al. 2012). Microarray analysis of the PPARβ-depleted islets showed that PPARα and -γ were slightly upregulated and that the amount of transcripts coding for peroxisomal genes was affected by the absence of PPARβ (Iglesias et al. 2012). We speculate that in the case of increased nutritional lipid uptake and consequent overload of the mitochondrial fatty acid transport due to saturated CPT1, heightened cytoplasmic lipids might activate PPARs and stimulate peroxisomal lipid transport and β-oxidation. Therefore, peroxisomal lipid transport and β-oxidation might be involved in a peroxisome/PPAR-feedback loop to control lipid homeostasis (Colasante et al. 2015). The compartmentalisation of the detoxification from H_2_O_2_ and of fatty acid degradation predisposes peroxisomes as an attractive target for further studies concerning the establishment of adult pancreas homeostasis as well as their possible involvement in the development of type II diabetes during ageing.

## Supplementary Information

Below is the link to the electronic supplementary material.

Supplementary file1 (TIF 11563 KB)

Supplementary file2 (TIF 4902 KB)

Supplementary file3 (TIF 2800 KB)

Supplementary file4 (DOCX 15 KB)

Supplementary file5 (DOCX 14 KB)

## Data Availability

The authors confirm that the data supporting the findings of this study are available within the article and its supplementary materials.
