# Supplementary figures and images for: Peroxisomes during postnatal development of mouse endocrine and exocrine pancreas display cell-type- and stage-specific protein composition
Source: Cell Tissue Res. 2023 May 1;393(1):63–81. doi: 10.1007/s00441-023-03766-6 (PMC10313850; doi:10.1007/s00441-023-03766-6)

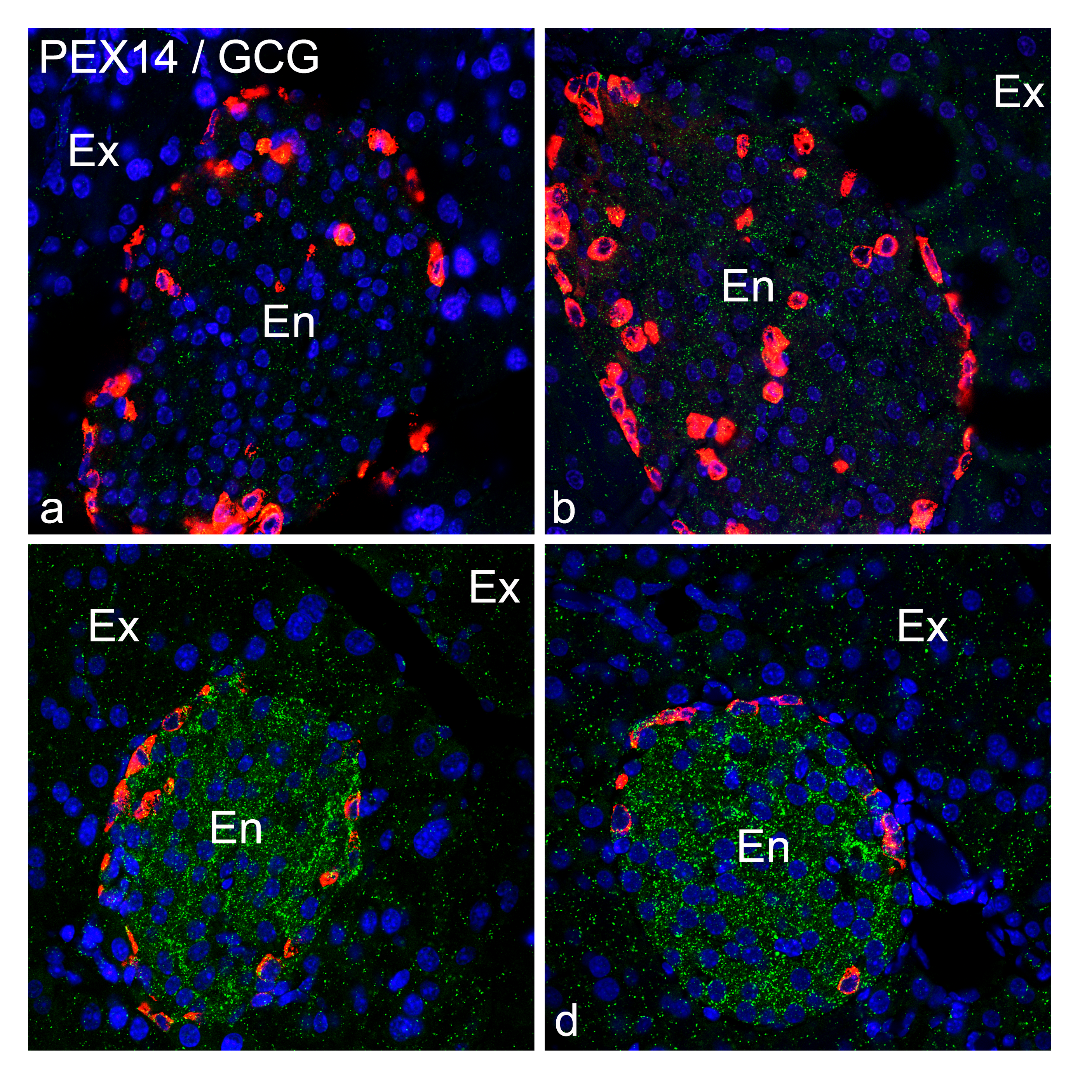

Supplement: Supplementary file 1 — Supplementary file1 (TIF 11563 KB) [file 441_2023_3766_MOESM1_ESM.tif]

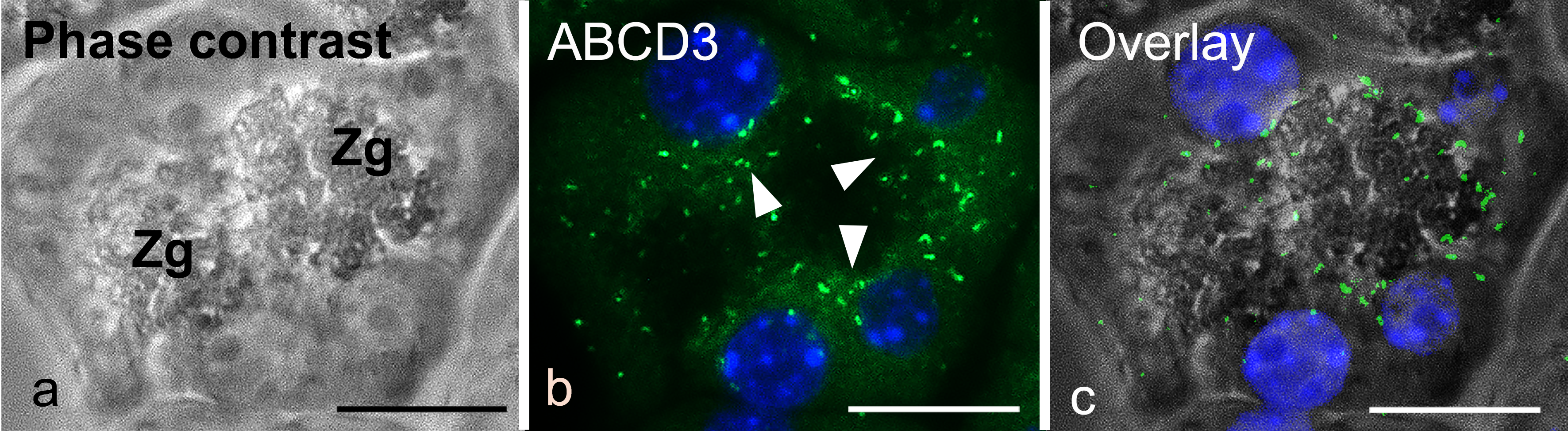

Supplement: Supplementary file 2 — Supplementary file2 (TIF 4902 KB) [file 441_2023_3766_MOESM2_ESM.tif]

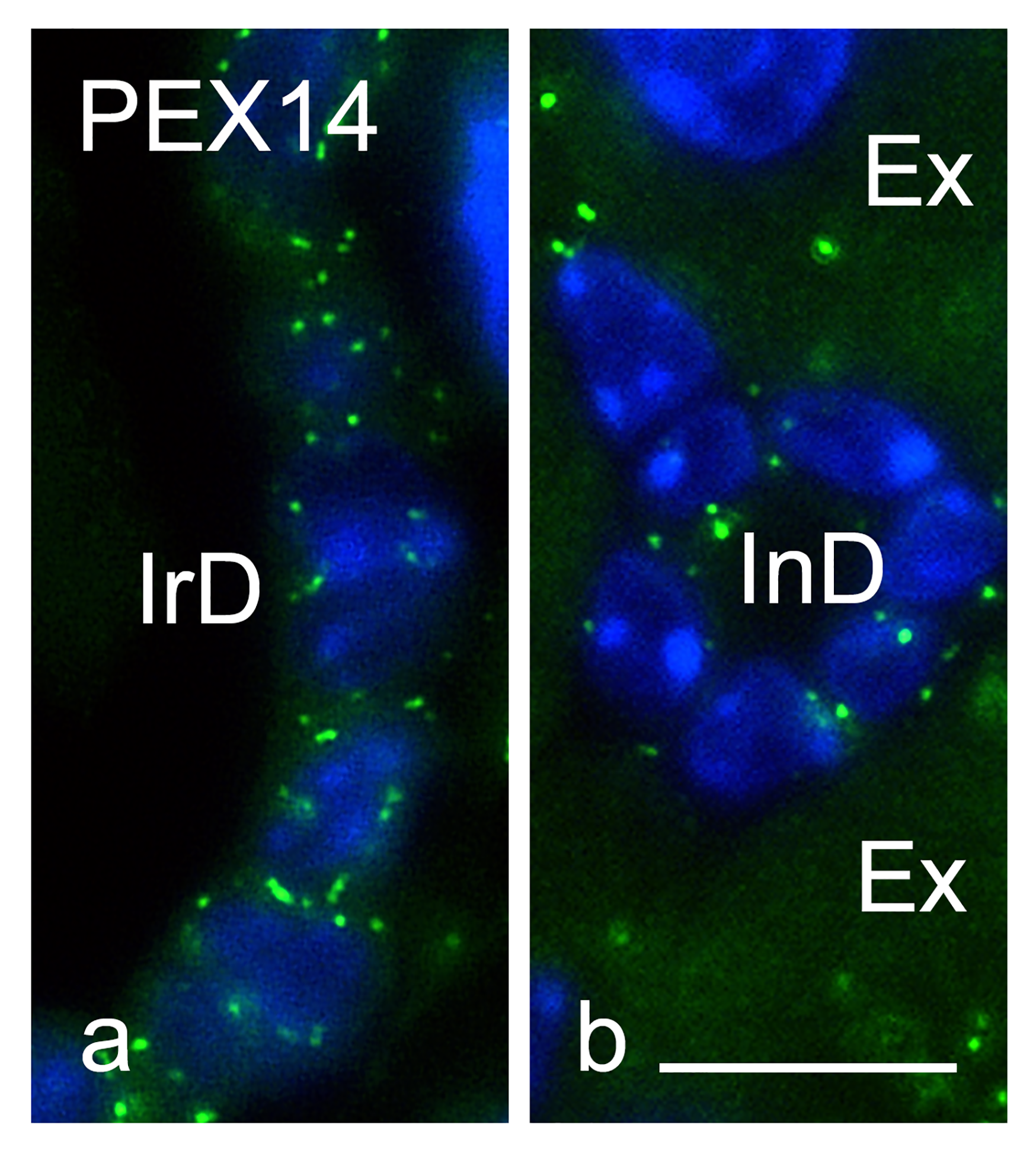

Supplement: Supplementary file 3 — Supplementary file3 (TIF 2800 KB) [file 441_2023_3766_MOESM3_ESM.tif]
