# Supplementary material for: Peroxisomes during postnatal development of mouse endocrine and exocrine pancreas display cell-type- and stage-specific protein composition
Source: Cell Tissue Res. 2023 May 1;393(1):63–81. doi: 10.1007/s00441-023-03766-6 (PMC10313850; doi:10.1007/s00441-023-03766-6)
Supplement: Supplementary file 4 — Supplementary file4 (DOCX 15 KB) [file 441_2023_3766_MOESM4_ESM.docx]

| Antigen | Host | Dilution (IF) | Dilution (WB) | Supplier |
| --- | --- | --- | --- | --- |
| ABCD3 | Rabbit | 1:1000 | 1:500 | Abcam |
| ACOX1 | Rabbit | 1:1000 | 1:10000 | Abcam |
| CAT | Rabbit | 1:1000 | 1:2000 | Gift from Prof. Denis Crane’s group‡ |
| GAPDH | Mouse |  | 1:10000 | HyTest Ltd |
| GCG | Mouse | 1:2000 |  | Sigma-Aldrich |
| Insulin + Proinsulin | Mouse | 1:2000 |  | Abcam |
| MFP2 | Rabbit | 1:5000 |  | Molecular Probes |
| PP | Rat | 1:1500 |  | Dako |
| PEX3 | Rat |  | 1:300 | Prof. Baumgart-Vogt’s group* |
| PEX14 | Rabbit | 1:2000 | 1:30000 | Gift from Prof. Crane’s group‡ |
| PEX19 | Rabbit |  | 1:10000 | Prof. Baumgart-Vogt’s group* |
| SST | Rat | 1:125 |  | RandD Systems |

* Institute for Anatomy and Cell Biology, JLU, Gießen, GE; ‡ School of Biomolecular and Physical Sciences, Griffith University, Brisbane, Qld, AUS. Abbreviations: ABCD3: ATP-binding cassette transporter, sub-family D, member 3; ACOX1: acyl-coenzyme a oxidase 1, palmitoyl; CAT: catalase; GAPDH: glyceraldehyde 3-phosphate dehydrogenase; GCG: glucagon; MFP2: multifunctional protein 2; PP: pancreatic polypeptide; PEX3, 14 and 19: peroxisomal biogenesis factor 3, 14 and 19; SST: somatostatin; IF: immunofluorescence; WB: Western blot
