# Supplementary material for: Peroxisomes during postnatal development of mouse endocrine and exocrine pancreas display cell-type- and stage-specific protein composition
Source: Cell Tissue Res. 2023 May 1;393(1):63–81. doi: 10.1007/s00441-023-03766-6 (PMC10313850; doi:10.1007/s00441-023-03766-6)
Supplement: Supplementary file 5 — Supplementary file5 (DOCX 14 KB) [file 441_2023_3766_MOESM5_ESM.docx]

| Antibody | Host | Dilution | Supplier |
| --- | --- | --- | --- |
| Western blotting | | | |
| Anti-mouse IgG horseradish peroxidase conjugate | Donkey | 1:50000 | Jackson Immuno ResearchA |
| Anti-rabbit IgG horseradish peroxidase conjugate | Donkey | 1:10000 | Jackson Immuno Research |
| Anti-rat IgG Immun-StarTM-AP | Goat | 1:4000 | Biorad |
| Immunofluorescence | | | |
| Anti-rabbit IgG Alexa Fluor 488 | Donkey | 1:300 | Molecular Probes |
| Anti-mouse IgG Texas Red | Horse | 1:300 | Vector Laboratories |
| Anti-rat Cy3 | Donkey | 1:400 | Dianova |
| Counterstaining of nuclei | | | |
| TO-PRO-3 Iodide | 1:750 | | Molecular Probes |
| Hoechst 33342 | 1:750 | | Sigma |
